# Cadherin-11 Mediates Contact Inhibition of Locomotion during *Xenopus* Neural Crest Cell Migration 

**DOI:** 10.1371/journal.pone.0085717

**Published:** 2013-12-31

**Authors:** Sarah F. S. Becker, Roberto Mayor, Jubin Kashef

**Affiliations:** 1 Zoological Institute, Cell- and Developmental Biology, Karlsruhe Institute of Technology (KIT), Karlsruhe, Germany; 2 Department of Cell and Developmental Biology, University College London, London, United Kingdom; University of Toledo, United States of America

## Abstract

Collective cell migration is an essential feature both in embryonic development and cancer progression. The molecular mechanisms of these coordinated directional cell movements still need to be elucidated. The migration of cranial neural crest (CNC) cells during embryogenesis is an excellent model for collective cell migration *in vivo*. These highly motile and multipotent cells migrate directionally on defined routes throughout the embryo. Interestingly, local cell-cell interactions seem to be the key force for directionality. CNC cells can change their migration direction by a repulsive cell response called contact inhibition of locomotion (CIL). Cell protrusions collapse upon homotypic cell-cell contact and internal repolarization leads to formation of new protrusions toward cell-free regions. Wnt/PCP signaling was shown to mediate activation of small RhoGTPase RhoA and inhibition of cell protrusions at the contact side. However, the mechanism how a cell recognizes the contact is poorly understood. Here, we demonstrate that *Xenopus* cadherin-11 (Xcad-11) mediated cell-cell adhesion is necessary in CIL for directional and collective migration of CNC cells. Reduction of Xcad-11 adhesive function resulted in higher invasiveness of CNC due to loss of CIL. Additionally, transplantation analyses revealed that CNC migratory behaviour *in vivo* is non-directional and incomplete when Xcad-11 adhesive function is impaired. Blocking Wnt/PCP signaling led to similar results underlining the importance of Xcad-11 in the mechanism of CIL and directional migration of CNC.

## Introduction

The cranial neural crest is a highly motile and multipotent cell population specific for vertebrates giving rise to a variety of craniofacial cell types such as cartilage, bones, melanocytes and elements of the peripheral nervous system [1-3]. At early gastrulation the CNC are induced at the border between the neural plate and epidermis by defined levels of Wnt, BMP, FGF, retinoic acid and Notch signaling [4,5]. In *Xenopus*, CNC cells emigrate shortly before neural tube closure from the anterior neural plate and migrate ventrally on distinct routes into the pharyngeal pouches. They start migrating first as a cohesive sheet and later disseminate into single cells making them an excellent model for both collective migration and cancer metastasis [6,7]. 

The directional migration of CNC is driven by a synergy of different mechanisms such as contact inhibition of locomotion (CIL), collective chemotaxis and coattraction [8-11]. CIL was defined as “the stopping of the continual locomotion of a cell in the same direction after collision with another cell” [12]. On the molecular level, CIL acts by recruiting the Wnt/PCP mediator Dishevelled (Dsh) to the membrane resulting in the local activation of the small GTPase RhoA at sites of cell-cell contact. This leads to intracellular polarization of the cell by generating an antagonistic RhoA-Rac1 gradient and the formation of new protrusions at the opposite side of cell-cell contact in a Rac1 dependent manner [8,13,14]. By this, CIL leads to high dispersion of single migrating CNC cells. Mutual coattraction acts as a counterbalancing mechanism repolarizing the cells towards each other via the complement fragment C3a [10]. In collective chemotaxis, the chemokine Sdf1 stabilizes CNC cell protrusions in a contact-dependent manner [9]. Taken together, local cell-cell interactions enable the cells to self-organize and to migrate directionally and collectively. 

For the recognition of cell-cell contact in CIL adhesion molecules such as cadherins were proposed [15,16]. Among them, N-Cadherin is expressed during CNC cell migration and promotes CNC migration *in vivo*. FRET analysis revealed that inhibition of Rac1 at the contact side is N-cadherin dependent [9]. The mesenchymal cadherin-11 is also expressed and involved in CNC migration [17-19], and thus, might promote CIL. Furthermore, up-regulation of the human homolog OB-cadherin is involved in tumor progression [20-22] and inflammatory and rheumatic arthritis [23,24]. This indicates that cadherin-11 can stimulate migration in addition to its cell-cell adhesive function. During *Xenopus* development, Xcad-11 expression starts when CNC cells acquire motility [17,25] and both Xcad-11 gain- and loss-of-function inhibited CNC migration [18,19]. Interestingly, Xcad-11 regulates protrusive activity via binding GEF-Trio and thereby modulating small RhoGTPases [19]. Here, we show that Xcad-11 mediated cell-cell adhesion is necessary for CIL and directional migration of CNC.

## Methods

### Ethics Statement

All animal studies were performed in strict accordance with German Animal Welfare legislation. All protocols and ethical evaluation were approved by the Institutional Animal Welfare Officer of the Karlsruhe Institute of Technology, and necessary licenses were obtained from the Regierungspraesidium Karlsruhe, Germany (the regional license granting body; permit numbers: 35-9185.81/G-27/10). Necessary anesthesia was performed under MS-222 and all efforts were made to minimize suffering.

### Constructs

The dominant negative construct for the adhesive function of Xcad-11 (dn-Xcad-11) was generated by site directed mutagenesis of W2A, W4A and point mutations of the QAV motif to LKG in full length Xcad-11. Dsh(DEP+), full-length Xcad-11 (fl-Xcad-11), dnRhoA, GAP43-mcherry, GAP43-GFP and H2B mcherry were published previously [19,26]. Xcad-11 morpholino antisense oligonucleotide (Xcad-11 MO) was designed as previously characterized [19] and purchased from Gene Tools, LLC (Philomath, OR, USA).

### Embryo manipulation, Cartilage staining, Whole mount in situ hybridization

Handling of *Xenopus laevis* embryos, CNC transplantations, CNC explants and cartilage stainings were performed as described previously [19]. Except for dnRhoA all constructs were transcribed *in vitro* into mRNA according to manufacture’s description (Ambion Inc., USA). 500 pg of the RNA constructs were injected into the D1 blastomere of eight-cell stage embryos, whereas for dnRhoA 10 pg DNA were used. For reconstitution experiments 100 pg of fl-Xcad-11 or dn-Xcad-11 RNA were injected together with 8 ng Xcad-11-MO, respectively. Whole mount *in situ* hybridization (ISH) was performed as described earlier [27]. Digoxigenin-labeled antisense RNA probes were generated according to manufacture’s description (Roche Diagnostics GmbH, Germany).

### Confrontation assay, Collision assay

CIL assays were performed as described [8], with slight modifications to the MatLab analysis script (see Figure S1). For collision assay, CNC cells were dissociated for three minutes with 0.3 mM EGTA in Danilchiks buffer (lacking CaCl_2_) and cultivated on fibronectin-coated chamber slides as described previously [8]. Live cell images were taken with Axio Observer.Z1 spinning disc confocal microscope with 5x plan apochromate NA 0.16 air objective, 10x plan apochromate NA 0.45 air objective and 63x plan apochromate NA 1.4 oil objective using AxioVision 4.8.2 software (Zeiss, Jena).

## Results and Discussion

### Depletion of Xcad-11 adhesive function blocks CNC migration *in vivo*

In CIL, non-canonical Wnt/PCP signaling and activation of RhoA at local cell-cell contact sites are necessary for cell polarity and thus directional migration [8]. However, the molecules mediating the cell-cell contact between two colliding cells and the molecular mechanisms how CIL components are localized at the membrane of the cell-cell contact side are poorly understood. Since Xcad-11 was shown to bind GEF-Trio via its cytoplasmic domain and to act upstream of the small RhoGTPases [19], we hypothesized, that this cell-cell adhesion molecule might be involved in CIL. 

**Figure 1:**
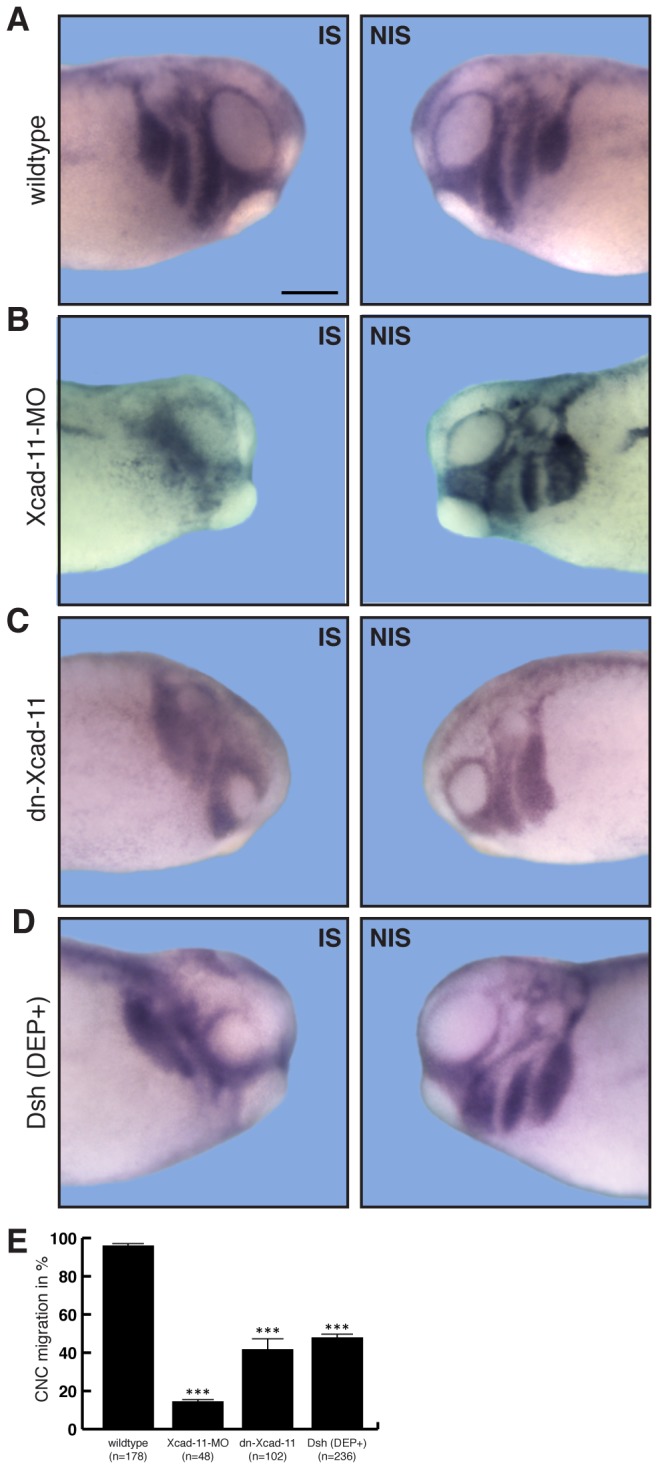
Depletion of Xcad-11 adhesive function blocks CNC migration *in vivo*. Lateral view of *Xenopus* CNC at stage 26, analysed by whole-mount ISH for the specific CNC marker AP-2α. Left column: Injected side (IS). Right column: Non-injected side (NIS). (A) Wildtype CNC cells migrated in defined streams into the pharyngeal pouches. (B) Xcad-11-MO injected embryos show AP-2α staining only at the dorsal part of the embryo, indicating incomplete CNC migration compared to NIS. (C) Overexpression of dn-Xcad-11 and (D) Dsh(DEP+) both showed incomplete and fused CNC hyoidal and branchial migration streams. Percentage of complete CNC migration given in (E) with n = number of embryos. Error bar shows standard error. (***) Significance to wildtype with p<0.005 after student’s T-Test. Scale bar, 250 µm.

To test this hypothesis, we generated a dominant negative construct (dnXcad-11), blocking the adhesive function of Xcad-11. This construct contains amino acid substitutions in the homophilic binding pocket including the two tryptophanes (W2A, W4A) and an altered QAV motif (LKG) within the first extracellular domain that are necessary for Xcad-11 trans-interactions [28,29]. *In situ* hybridization for AP2αa marker for CNC, revealed that wildtype cells were able to migrate into all arches (96% migration of CNC; n= 178; Figure 1A, E). In contrast, embryos injected with Xcad-11MO showed a severe migration phenotype. The AP2α signal could only be detected at the dorsal part adjacent to the brain but not in pharyngeal pouches (86% loss of migration; n=48; Figure 1B, E; injected side (IS), non-injected side (NIS); [19]). Overexpression of dn-Xcad-11 led to inhibition of migration in the hyoidal and branchial arches in 58% (n=102) of the injected embryos. The altered expression pattern of the CNC marker gene AP2α revealed that migration was incomplete and migration streams were fused (Figure 1C, E). Because contact mediated localization of Dsh to the membrane is necessary for CNC migration and CIL [8,26] we analysed whether overexpression of the DEP domain of Dsh (Dsh(DEP+)) inhibits CNC migration in a similar manner. Indeed, Dsh(DEP+) which specifically blocked Wnt/PCP pathway without interfering with canonical Wnt signaling inhibited migration in 52% (n=236) of the injected embryos (Figure 1D, E). Thus, Xcad-11 adhesive function and Wnt/PCP are essential for proper CNC migration. 

**Figure 2:**
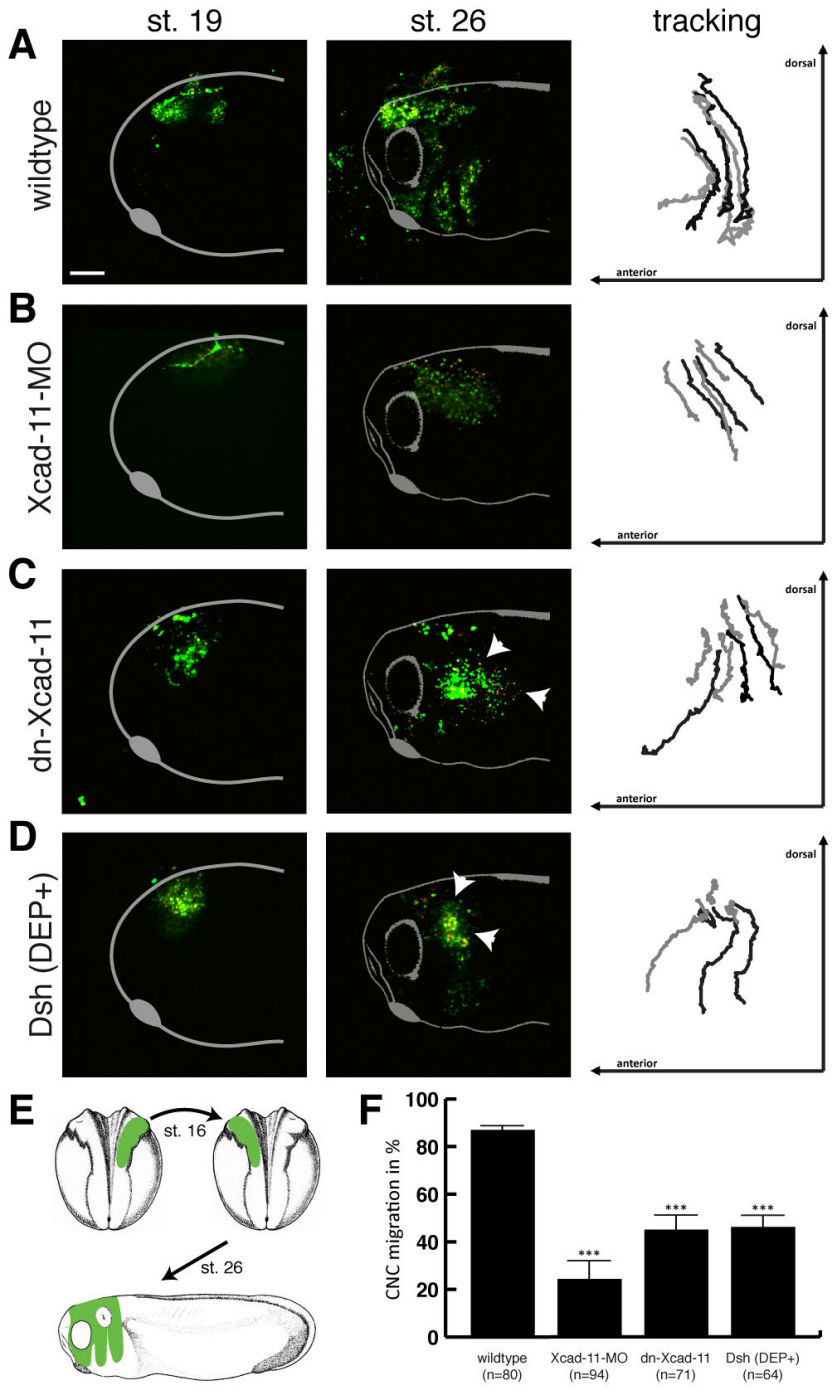
Xcad-11 is required for directional CNC migration *in vivo*. CNC transplants. First column: Lateral view on transplanted GFP-labelled *Xenopus* CNC before migration at stage 19. Second column: Lateral view on transplanted GFP-labelled *Xenopus* CNC after migration at stage 26. Third column: Tracking analysis of six to seven cells by H2B-cherry labelled nuclei during CNC migration (stage 19-26). Anterior is to the left and dorsal to the top. (A) Wildtype grafts showed normal migration. (B) Grafts coinjected with Xcad-11-MO were unable to migrate into the pharyngeal pouches. (C, D) Overexpression by coinjection of dn-Xcad-11 and Dsh(DEP+), respectively, led to disorientated and not directional CNC migration. (E) Schematic illustration of the transplantation assay. Percentage of complete CNC migration given in (F) with n = number of transplanted embryos. Error bar shows standard error. (***) Significance to wildtype with p<0.005 after student’s T-Test. Scale bar, 200 µm.

Marker gene analyses by *in situ* hybridization gives always only a snapshot of developing embryos, but is not suitable to analyse dynamics in cell migration. Therefore, we performed *in vivo* time-lapse and tracking analysis in CNC transplanted embryos. To follow and track CNC cells during their migration *in vivo* we coinjected GAP43-GFP (as a membrane marker) and H2B mcherry (as a nuclei marker). Wildtype CNC grafts exhibited directional migration towards the pharyngeal pouches (87%; n=80; Figure 2A, E, F; Movie S1). In contrast, grafts from Xcad-11 morphants were not able to enter into the hyoidal and branchial arches, although they start to migrate as a collective sheet (76% loss of migration; n=94; Figure 2B, F; Movie S2). Overexpression of dn-Xcad-11 led in 55% (n=71) of the embryos to incomplete migration. CNC cells were disorientated and unable to migrate directionally (Figure 2C, F; white arrowheads; Movie S3). Blocking Wnt/PCP pathway by overexpression of Dsh(DEP+) led to 54% of embryos with incomplete migration (Figure 2D, F; white arrowheads). Although some CNC were able to find their final destination in the pharyngeal pouches, most of the cells did not migrate directionally similar to CNC cells lacking Xcad-11 mediated adhesion (Movie S3 and S4). To exclude that incomplete CNC migration in the grafted embryos is due to a developmental delay we performed transplantation experiments and analysed CNC migration at later developmental stages 33/34 (Figure S2). Wildtype CNC grafts exhibited normal migration (Figure S2A). As expected, Xcad-11 morphant CNC are still not able to migrate into the pharyngeal pouches (Figure S2B). Also transplanted CNC cells injected with dn-Xcad-11 or Dsh(DEP+) showed incomplete migration (Figure S2C, D; white arrowheads). CNC cells migrated disorientated or fused branchial arches were observed. CNC differentiate to cartilage and bone structures of the embryonic head. Therefore, we examined additionally cartilage formation (Figure S2). Wildtype embryos exhibited bilateral symmetric cartilage structures (Figure S2A) whereas Xcad-11 morphant embryos displayed severe cartilage defects including loss of meckel’s cartilage and reduced posterior cartilage structures (Figure S2B). Loss of bilateral symmetry and reduced cartilage structures were also observed for overexpression of dn-Xcad-11 and Dsh(DEP+) (Figure S2C, D).

Taken together, we conclude that CNC cells need Xcad-11 facilitated cell-cell adhesion for collective migration and CIL. 

### Loss of Xcad-11 adhesive function increases CNC invasiveness

**Figure 3:**
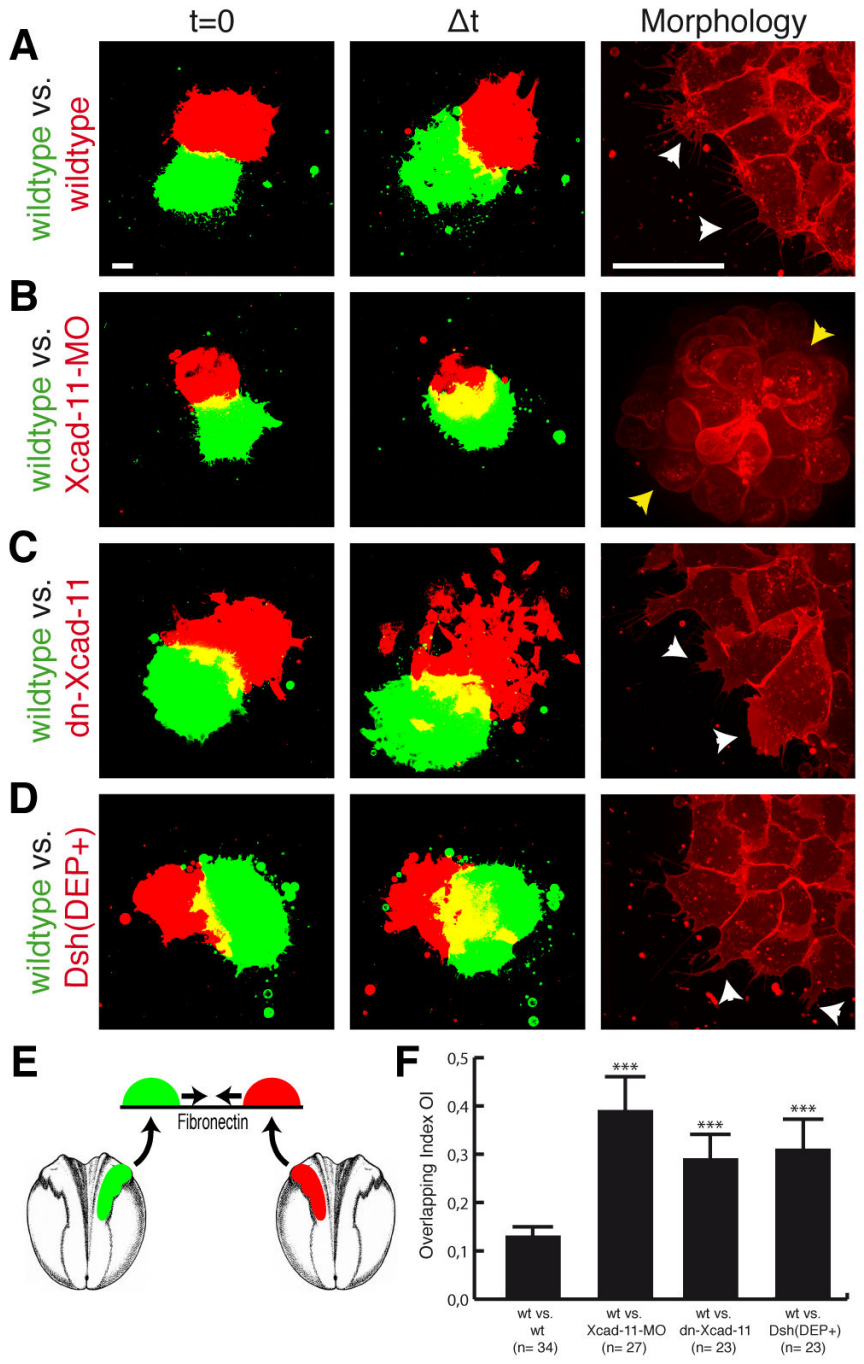
Loss of Xcad-11 adhesive function increases CNC invasiveness *in vitro*. Confrontation assay. First column: Confronted explants at time point t=0. Second column: Confronted CNC explants at time point of highest invasion Δt. Third column: Morphology of CNC cells. Except for (A) wildtype vs. wildtype, yellow overlapping area increased strongly in (B) Xcad-11 morphant, (C) dn-Xcad-11 and (D) Dsh(DEP+) overexpressing CNC cells reflecting invasiveness of the tissues. Xcad-11 depleted cells displayed blebbing (yellow arrowheads in (B)) in contrast to protrusion formation of CNC cells in other approaches (white arrowheads in (A, C, D)). (E) Schematic illustration of the confrontation assay. Average Overlapping Index (OI) given in (F) with n = number of confrontations (wt: wildtype). Error bar shows standard error. (***) Significance to wildtype vs. wildtype with p<0.001 after student’s T-Test. Scale bar, 50 µm.

The similarity of migration defects observed for the loss of Xcad-11 adhesive function and loss of Wnt/PCP signaling prompted us to speculate that Xcad-11 is the molecule mediating cell-cell contacts in CIL. If so, dnXcad-11 should prevent contact inhibition of locomotion in a similar manner as Dsh(DEP+). To test this hypothesis we performed explant confrontation assays *in vitro* ([8], Figure 3E). The quantification by the overlapping index (OI) compares the overlapping area of two confronted explants to the normalized area of one explant at the time point of highest invasion Δt (see Figure S1). Loss of CIL was measured by higher invasion and increase of OI. Fluorescently labelled wildtype CNC explants showed normal CNC cell morphology with dynamic filopodia and lamellipodia formation (Figure 3A, white arrowheads). These explants did not invade each other due to homotypic CIL, as shown by an OI = 13.1% (n=34; Figure 3A, F; Movie S5). Depletion of Xcad-11 via MO knockdown led to a significantly higher invasion of the explants (OI = 39.7%; n=27; Figure 3B, F; Movie S6). Given that the morphant CNC cells were unable to form cell protrusions (Figure 3B; yellow arrowheads; [19]), which are necessary for cell migration, the increased OI in the Xcad-11 depleted cells might also reflect that they failed to escape from the contact area. In contrast, CNC cells overexpressing dn-Xcad-11 were able to dynamically form cell protrusions (Figure 3C; white arrowheads). Strikingly, loss of Xcad-11 mediated adhesion increased explant invasiveness significantly with OI of 29.2% (n=23) indicating that Xcad-11 mediated cell-cell adhesion is indeed a prerequisite for CIL (Figure 3C, F; Movie S7). Since overexpression of dn-Xcad-11 could not exclude the presence of endogenous Xcad-11 and might give rise to misleading results we performed rescue experiments with fl-Xcad-11 or dn-Xcad-11 in a Xcad-11 MO knockdown background and analysed CIL by the confrontation assay. Fl-Xcad-11 injection restored cell protrusion formation and CIL (OI = 9.5%, n=10; Figure S3A, C; Movie S8) whereas dn-Xcad-11 coinjected CNC explants displayed an OI of 24.6% (n=10; Figure S3B, C; Movie S9) indicating a higher invasiveness as the wildtype. Additionally, we analysed the rescue capacity of fl-Xcad-11 and dn-Xcad-11 *in vivo* (Figure S3D-F). *In situ* hybridization for AP2α demonstrated that coinjection of fl-Xcad-11 could rescue CNC migration (74.9%; n=169; Figure S3D, F). In contrast, CNC cells injected with dn-Xcad-11 and Xcad-11 MO were not able to reach the pharyngeal pouches and displayed fused arches (63.4% loss of migration; n=115; Figure S3E, F). Compared to Xcad-11 morphants we could observe no significant rescue in dn-Xcad-11 coinjected embryos *in vivo*. Indeed, dn-Xcad-11 coinjected CNC cells were able to form cell protrusion and to migrate *in vitro* (Figure S3B; Movie S9), indicating that their motility was not disturbed, but they lost the ability to migrate directional into the pharyngeal pouches *in vivo* (Figure S3E). Therefore, we conclude that for directional CNC migration the adhesive function of Xcad-11 is essential.

Again, injection of Dsh(DEP+) phenocopied loss of Xcad-11 mediated cell-cell adhesion suggesting an important function of Xcad-11 in CIL (OI = 31.9%; n=23; Figure 3D, F; Movie S10). Since it was demonstrated that activated RhoA drives CIL and therefore CNC migration [8] we investigated the impact of RhoA activity. Thus, we injected a dominant negative RhoA construct (dn-RhoA) and performed the confrontation assay. Loss of RhoA activity increased explant invasion *in vitro* (OI = 21.4%; n=10; Figure S4A, B; Movie S11). This is in agreement with our previous data where inhibition of RhoA blocked CNC migration *in vivo* [19]. 

### Xcad-11 mediates repulsive response in colliding single CNC cells *in vitro*

**Figure 4:**
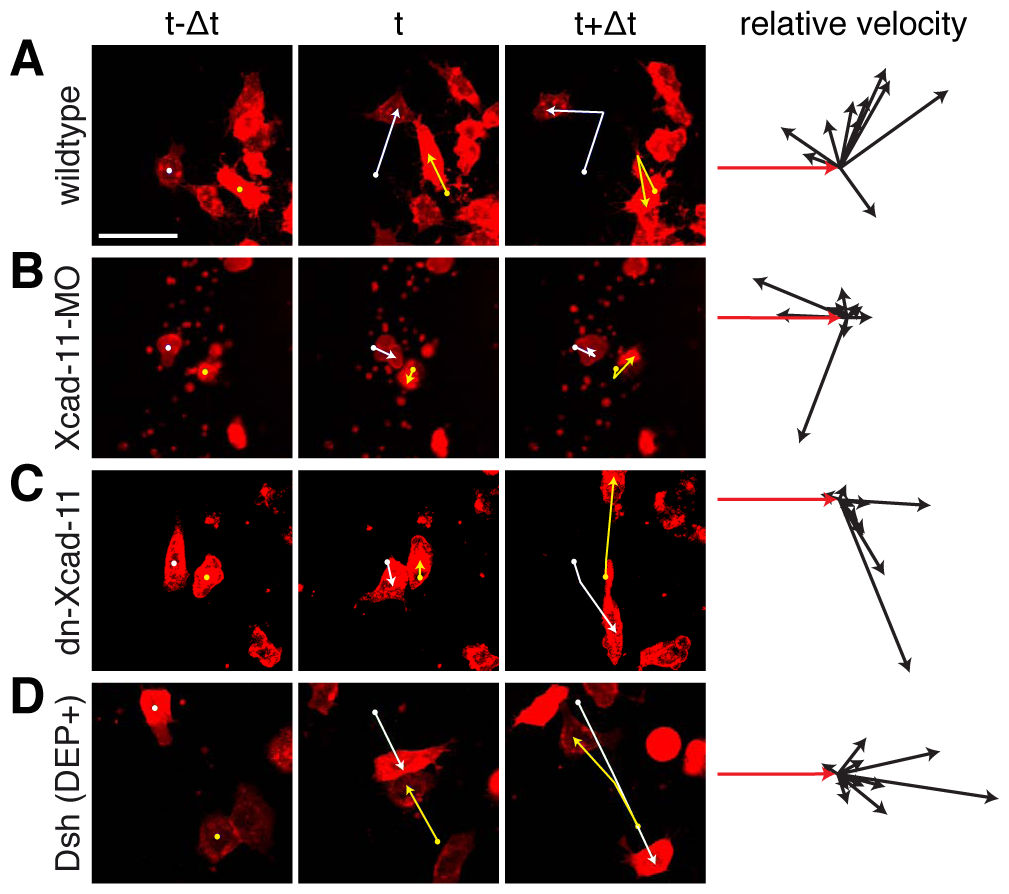
Xcad-11 mediates repulsive response in colliding in single CNC cells *in vitro*. Collision assay. First three columns: Single CNC cells before (t-Δ), during (t) and after (t+Δ) mutual contact with tracking. Fourth column: Relative velocity vectors with initial velocity vector (red, n = 10 collisions). (A) Only wildtype CNC cells showed change of direction. (B) Xcad-11-MO treated cells show random distribution. (C) dn-Xcad-11 and (D) Dsh(DEP+) overexpressing CNC cells displayed reduced repulsive response. Scale bar, 50 µm.

Based on the first definition of CIL [12] we performed collision assays to investigate single CNC cell migration behaviour [8]. As expected, wildtype CNC cells exhibited the typical CIL response by changing the direction of migration after collision in regard to the initial vector (in red) as demonstrated by relative velocity analyses (Figure 4A). Colliding Xcad-11-MO treated CNC cells displayed a physical bouncing effect with a random distribution of the velocity vectors. This might be due to the fact that these cells were both unable to form protrusions and migrate actively (Figure 4B). In contrast, collisions of two dn-Xcad-11 overexpressing cells led to reduced repulsive response. Here, nearly all CNC cells slid along each other and showed reduced change of direction upon contact, as compared with wildtype CNC cells (Figure 4C). Similar results were obtained for reconstitution experiments with coinjection of fl-Xcad-11 and dn-Xcad-11 (Figure S3G, H). Disturbing PCP pathway by overexpression of Dsh(DEP+) led to no change of direction upon contact (Figure 4D; [8]). Interestingly, blocking Rock, a downstream effector of RhoA, led to a complete loss of CIL [8]. Again, CNC with loss of Xcad-11 mediated adhesion mimics the positive control of inhibited CIL by blocking Wnt/PCP pathway. 

In this present study we showed that Xcad-11 is a key molecule to mediate cell-cell adhesion for CIL. CIL has been described as having two phases: (1) *cell-cell adhesion* followed by a collapse of cell protrusions at the contact site and (2) repolarization of the cells in a RhoA dependent manner, which leads to *repulsion* and migration of cells in the opposite direction [8,15]. However, the precise mechanism of contact mediation and how Xcad-11 mediated cell-cell contacts influence RhoA activation at the contact side still needs to be elucidated. Interestingly, a defined level of Xcad-11 mediated cell-cell adhesion is important for proper CNC migration, since overexpression of high amounts of fl-Xcad-11 or its extracellular domain resulted in increased adhesion and disturbed CNC migration [18]. This indicates together with our present data that Xcad-11 modulated cell-cell adhesion is needed during CNC migration. 

The extracellular domain of Xcad-11 is cleaved by the metalloproteinase ADAM-13, which reduces cell-cell adhesion. Conversely, loss of ADAM-13 activity led to enhanced Xcad-11 mediated adhesion and blocked CNC migration *in vivo* [30]. Thus, ADAM-13 might reduce Xcad-11 mediated cell-cell adhesion at the contact site and therefore possibly enforcing single cell migration needed for CIL. 

During CIL the activation of RhoA can be observed at the cell-cell contact site [8]. The interaction of the cytoplasmic domain of Xcad-11 with the GEF-Trio [19] might provide the molecular mechanism for this RhoA activation. GEF-Trio has two GEF domains: GEF1 activating Rac1 and GEF2 regulating RhoA activity [31,32]. Furthermore, it was demonstrated that GEF-Trio is involved in regulation of axon guidance and neuronal migration [33]. Interestingly, we recently demonstrated that GEF-Trio is localized at cell-cell contacts and promote CNC migration *in vivo* [34]. Hence, Xcad-11 mediated localization of GEF-Trio to the cell-cell contact could lead to an activation of RhoA at the contact site resulting in the repolarization and migration of CNC cells in the opposite direction. However, further studies on the Xcad-11/GEF-Trio complex are needed to elucidate its role in CIL.

Cadherins are connected by different mechanisms to small RhoGTPases important for generating signalling cascades that control cell-cell adhesion and cell polarity [35]. Recently, it has been shown that N-cadherin regulates CNC migration through CIL [9]. Blocking N-Cadherin function through an antibody both increased CNC invasiveness and inhibited single cell repulsive response [9]. N-cadherin inhibition led to an increase of Rac1 activity at the cell-cell contact probably due to a lack of RhoA activation downstream of the Wnt/PCP pathway [9]. However, the precise mechanism of interaction between N-cadherin and Wnt/PCP during CIL remains to be characterized. Thus, it will be important to analyse how a possible interaction of N-Cadherin and/or Xcad-11 could promote CIL via downstream factors like Dsh, GEF-Trio and the small RhoGTPases. Since inhibiting N-cadherin and Xcad-11 separately affected CIL we suppose that a global level of cell-cell adhesion is required for CIL, therefore decreasing N-cadherin or Xcad-11 affects this level of adhesion impairing CIL.

We propose that Xcad-11 adhesive function is necessary to recruit CIL components to the membrane and that contact recognition acts via Xcad-11 mediated cell-cell adhesion. The role of cadherin-11 in CIL may be of particular interest. Many cancer cells display loss of heterotypic CIL towards healthy cells [36-38]. Since cadherin-11 is responsible for enhancing invasion of malignant cells into healthy tissues in prostate and breast cancer [20,21,39] our findings will help to further understand cell invasion in different diseases. 

## Supporting Information

Figure S1 **Analysis of explant confrontation assay.** Using a newly developed MatLab script we analysed the overlapping index OI at the time point of highest overlapping Δt. We measured the size of the overlapping area c (yellow) and compared c to the size of the normalized area of the single explants 2d^2^ (green or red). We chose to normalize the size of explants dependent on the contact border in order to be more independent of the different sizes of the explants. Therefore, 2d^2^ includes the area of explant within two squares based on the contact border. The mean values of both OI for red and green explants were taken for all confrontations of one approach and averaged.
(TIF)

Figure S2 **Blocking Xcad-11 mediated cell-cell adhesion leads to incomplete CNC migration also in advanced stages.** CNC transplants at stage 33/34. First column: Lateral view on transplanted GFP-labelled *Xenopus* CNC in brightfield and FITC channel. Second column: Lateral view on transplanted GFP-labelled *Xenopus* CNC in FITC channel. Anterior is to the left and dorsal to the top. (A) Wildtype grafts showed normal migration. (B) Grafts coinjected with Xcad-11-MO were unable to migrate into the pharyngeal pouches. (C, D) Overexpression by injection of dn-Xcad-11 and Dsh(DEP+), respectively, led to disorientated and not directional CNC migration (white arrowheads).
Cartilage staining at stage 45. Third column: (A) Wildtype embryos showed bilateral symmetric cartilage structures. (B) Xcad-11 morphant embryos displayed severe cartilage defects including loss of meckel’s cartilage and reduced posterior cartilage structures. (C, D) Overexpression of dn-Xcad-11 or Dsh(DEP+), respectively, led to loss of bilateral symmetry and reduced cartilage structures. Asterisks indicate injected side. Scale bar, 250 µm.
(TIF)

Figure S3 **Reconstitution experiments with fl-Xcad-11 and dn-Xcad-11 show importance of Xcad-11 mediated cell-cell adhesion in CIL.** (A-C) Confrontation assay. First column: Confronted explants at time point t=0. Second column: Confronted CNC explants at time point of highest invasion Δt. Third column: Morphology of CNC cells. (A) Coinjection of fl-Xcad-11 could rescue CIL, whereas (B) coinjection of dn-Xcad-11 displayed an increased yellow overlapping area. (A, B) Protrusion formation could be restored in both cases (white arrowheads). (C) Average Overlapping Index (OI) with n = number of confrontations (wt: wildtype). Error bar shows standard error. (***) Significance with p<0.005 after student’s T-Test. Scale bar, 50 µm.
(D-F) Lateral view of *Xenopus* CNC at stage 26, analysed by whole-mount ISH for the specific CNC marker AP-2α. Left column: Injected side (IS). Right column: Non-injected side (NIS). (D) Coinjection of fl-Xcad-11 could rescue CNC migration, whereas (E) coinjection of dn-Xcad-11 showed incomplete and fused CNC hyoidal and branchial migration streams compared to NIS. Percentage of complete CNC migration given in (F) with n = number of embryos. Error bar shows standard error. (***) Significance with p<0.001 after student’s T-Test. Scale bar, 250 µm.
(G, H) Collision assay. First three columns: Single CNC cells before (t-Δ), during (t) and after (t+Δ) mutual contact with tracking. Fourth column: Relative velocity vectors with initial velocity vector (red, n = 10 collisions). (G) Coinjection of fl-Xcad-11 rescued repulsive response, whereas (H) coinjection of dn-Xcad-11 led to no change of direction. Scale bar, 50 µm.
(TIF)

Figure S4 **Loss of RhoA activity leads to loss of CIL.** (A, B) Confrontation assay. First column: Confronted explants at time point t=0. Second column: Confronted CNC explants at time point of highest invasion Δt. Third column: Morphology of CNC cells. (A) dnRhoA led to an increased yellow overlapping area, whereas protrusion formation was same as wildtype (white arrowheads). (B) Average Overlapping Index (OI) with n = number of confrontations (wt: wildtype). Error bar shows standard error. (***) Significance to wildtype vs. wildtype with p<0.005 after student’s T-Test. Scale bar, 50 µm.
(TIF)

Movie S1 **Time-lapse analysis of transplanted wildtype CNC labelled with GAP43-GFP and H2B mcherry.** Transplants were analysed from stage 19 to stage 26, when the CNC cells have reached their final destination. Anterior is to the left and dorsal to the top. Wildtype CNC cells showed complete migration into pharyngeal pouches. Images captured every ten minutes over a period of several hours as indicated in the movie and taken with Axio Observer.Z1 spinning disc confocal microscope using a 10x plan apochromate NA 0.45 air objective. Scale bar: 100 µm.
(MP4)

Movie S2 **Time-lapse analysis of transplanted CNC labelled with GAP43-GFP and H2B mcherry and coinjected with Xcad-11-MO.** Transplants were examined from stage 19 to stage 26. Anterior is to the left and dorsal to the top. Note that Xcad-11 morphant CNC cells displayed incomplete migration. CNC cells migrated as a cluster, but were unable to emigrate as single cells into distinct migration streams. Images captured every ten minutes over a period of several hours as indicated in the movie and taken with Axio Observer.Z1 spinning disc confocal microscope using a 10x plan apochromate NA 0.45 air objective. Scale bar: 100 µm.
(MP4)

Movie S3 **Time-lapse analysis of transplanted CNC labelled with GAP43-GFP and H2B mcherry and coinjected with dn-Xcad-11.** Transplants were investigated from stage 19 to stage 26. Anterior is to the left and dorsal to the top. CNC cells overexpressing dn-Xcad-11 showed incomplete and disorientated migration. Images captured every ten minutes over a period of several hours as indicated in the movie and taken with Axio Observer.Z1 spinning disc confocal microscope using a 10x plan apochromate NA 0.45 air objective. Scale bar: 100 µm.
(MP4)

Movie S4 **Time-lapse analysis of transplanted CNC labelled with GAP43-GFP and H2B mcherry and coinjected with Dsh(DEP)+.** Transplants were investigated from stage 19 to stage 26. Anterior is to the left and dorsal to the top. CNC cells overexpressing Dsh(DEP)+ displayed incomplete and disorientated migration. Images captured every ten minutes over a period of several hours as indicated in the movie and taken with Axio Observer.Z1 spinning disc confocal microscope using a 10x plan apochromate NA 0.45 air objective. Scale bar: 100 µm.
(MP4)

Movie S5 **Confronting GAP43-GFP-labeled wildtype CNC explant with GAP43-mcherry-labeled wildtype CNC explant led to low overlapping (yellow).** Images captured every three minutes over a period of several hours as indicated in the movie and taken with Axio Observer.Z1 spinning disc confocal microscope using a 10x plan apochromate NA 0.45 air objective. Scale bar: 50 µm.
(MP4)

Movie S6 **Confronting GAP43-GFP-labeled wildtype CNC explant with GAP43-mcherry-labeled CNC explant coinjected with Xcad-11-MO led to increased overlapping (yellow).** Images captured every three minutes over a period of several hours as indicated in the movie and taken with Axio Observer.Z1 spinning disc confocal microscope using a 10x plan apochromate NA 0.45 air objective. Scale bar: 50 µm.
(MP4)

Movie S7 **Confronting GAP43-GFP-labeled wildtype CNC explant with GAP43-mcherry-labeled CNC explant coinjected with dn-Xcad-11 led to increased overlapping (yellow).** Note that single dn-Xcad-11 CNC cells invaded into the other tissue. Images captured every three minutes over a period of several hours as indicated in the movie and taken with Axio Observer.Z1 spinning disc confocal microscope using a 10x plan apochromate NA 0.45 air objective. Scale bar: 50 µm.
(MP4)

Movie S8 **Confronting GAP43-GFP-labeled wildtype CNC explant with GAP43-mcherry-labeled CNC explant coinjected with Xcad-11-MO and fl-Xcad-11 led to low overlapping (yellow).** Images captured every three minutes over a period of several hours as indicated in the movie and taken with Axio Observer.Z1 spinning disc confocal microscope using a 10x plan apochromate NA 0.45 air objective. Scale bar: 50 µm.
(MP4)

Movie S9 **Confronting GAP43-GFP-labeled wildtype CNC explant with GAP43-mcherry-labeled CNC explant coinjected with Xcad-11-MO and dn-Xcad-11 led to increased overlapping (yellow).** Images captured every three minutes over a period of several hours as indicated in the movie and taken with Axio Observer.Z1 spinning disc confocal microscope using a 10x plan apochromate NA 0.45 air objective. Scale bar: 50 µm.
(MP4)

Movie S10 **Confronting GAP43-GFP-labeled wildtype CNC explant with GAP43-mcherry-labeled CNC explant coinjected with Dsh(DEP)+ led to increased overlapping (yellow).** Note that single Dsh(DEP+) CNC cells invaded into the other tissue. Images captured every three minutes over a period of several hours as indicated in the movie and taken with Axio Observer.Z1 spinning disc confocal microscope using a 10x plan apochromate NA 0.45 air objective. Scale bar: 50 µm.
(MP4)

Movie S11 **Confronting GAP43-GFP-labeled wildtype CNC explant with GAP43-mcherry-labeled CNC explant coinjected with dn-RhoA led to increased overlapping (yellow).** Images captured every three minutes over a period of several hours as indicated in the movie and taken with Axio Observer.Z1 spinning disc confocal microscope using a 10x plan apochromate NA 0.45 air objective. Scale bar: 50 µm.
(MP4)
