# Supplementary figures and images for: Cadherin-11 Mediates Contact Inhibition of Locomotion during Xenopus Neural Crest Cell Migration
Source: PLoS One. 2013 Dec 31;8(12):e85717. doi: 10.1371/journal.pone.0085717 (PMC3877381; doi:10.1371/journal.pone.0085717)

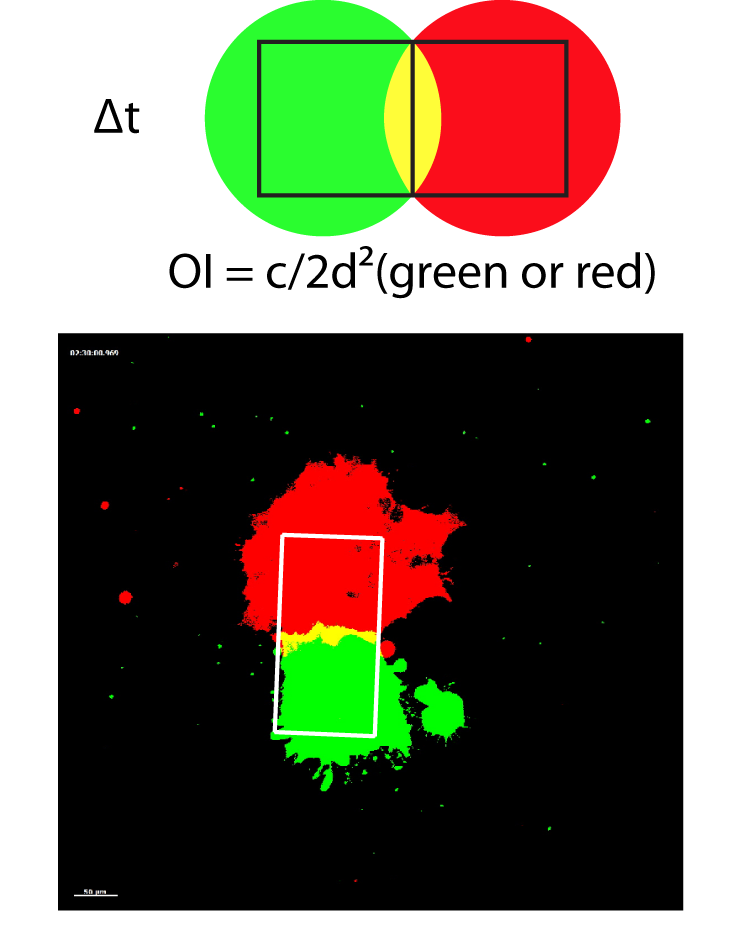

Supplement: Figure S1 — Analysis of explant confrontation assay. Using a newly developed MatLab script we analysed the overlapping index OI at the time point of highest overlapping Δt. We measured the size of the overlapping area c (yellow) and compared c to the size of the normalized area of the single explants 2d2 (green or red). We chose to normalize the size of explants dependent on the contact border in order to be more independent of the different sizes of the explants. Therefore, 2d2 includes the area of explant within two squares based on the contact border. The mean values of both OI for red and green explants were taken for all confrontations of one approach and averaged. (TIF) [file pone.0085717.s001.tif]

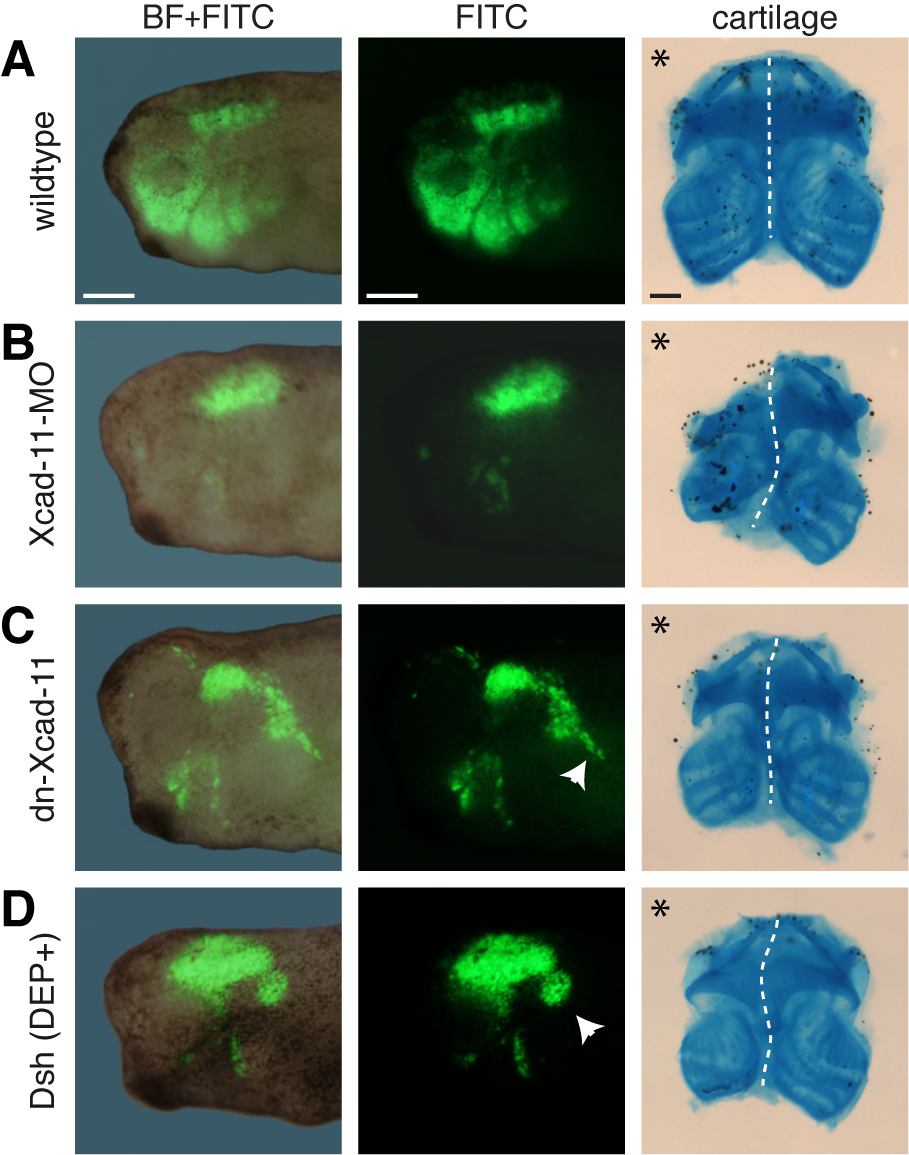

Supplement: Figure S2 — Blocking Xcad-11 mediated cell-cell adhesion leads to incomplete CNC migration also in advanced stages. CNC transplants at stage 33/34. First column: Lateral view on transplanted GFP-labelled Xenopus CNC in brightfield and FITC channel. Second column: Lateral view on transplanted GFP-labelled Xenopus CNC in FITC channel. Anterior is to the left and dorsal to the top. (A) Wildtype grafts showed normal migration. (B) Grafts coinjected with Xcad-11-MO were unable to migrate into the pharyngeal pouches. (C, D) Overexpression by injection of dn-Xcad-11 and Dsh(DEP+), respectively, led to disorientated and not directional CNC migration (white arrowheads). Cartilage staining at stage 45. Third column: (A) Wildtype embryos showed bilateral symmetric cartilage structures. (B) Xcad-11 morphant embryos displayed severe cartilage defects including loss of meckel’s cartilage and reduced posterior cartilage structures. (C, D) Overexpression of dn-Xcad-11 or Dsh(DEP+), respectively, led to loss of bilateral symmetry and reduced cartilage structures. Asterisks indicate injected side. Scale bar, 250 µm. (TIF) [file pone.0085717.s002.tif]

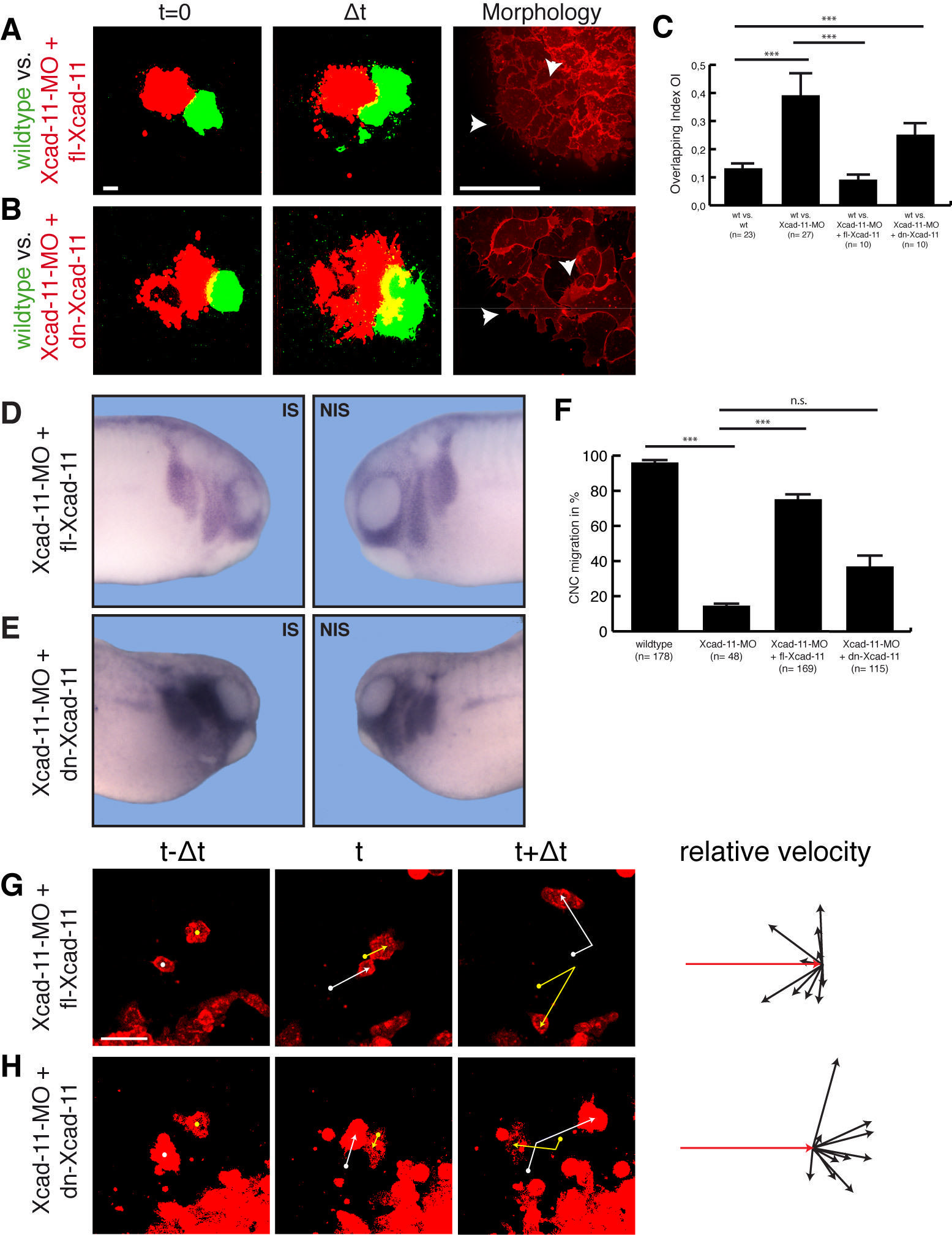

Supplement: Figure S3 — Reconstitution experiments with fl-Xcad-11 and dn-Xcad-11 show importance of Xcad-11 mediated cell-cell adhesion in CIL. (A-C) Confrontation assay. First column: Confronted explants at time point t=0. Second column: Confronted CNC explants at time point of highest invasion Δt. Third column: Morphology of CNC cells. (A) Coinjection of fl-Xcad-11 could rescue CIL, whereas (B) coinjection of dn-Xcad-11 displayed an increased yellow overlapping area. (A, B) Protrusion formation could be restored in both cases (white arrowheads). (C) Average Overlapping Index (OI) with n = number of confrontations (wt: wildtype). Error bar shows standard error. (***) Significance with p<0.005 after student’s T-Test. Scale bar, 50 µm. (D-F) Lateral view of Xenopus CNC at stage 26, analysed by whole-mount ISH for the specific CNC marker AP-2α. Left column: Injected side (IS). Right column: Non-injected side (NIS). (D) Coinjection of fl-Xcad-11 could rescue CNC migration, whereas (E) coinjection of dn-Xcad-11 showed incomplete and fused CNC hyoidal and branchial migration streams compared to NIS. Percentage of complete CNC migration given in (F) with n = number of embryos. Error bar shows standard error. (***) Significance with p<0.001 after student’s T-Test. Scale bar, 250 µm. (G, H) Collision assay. First three columns: Single CNC cells before (t-Δ), during (t) and after (t+Δ) mutual contact with tracking. Fourth column: Relative velocity vectors with initial velocity vector (red, n = 10 collisions). (G) Coinjection of fl-Xcad-11 rescued repulsive response, whereas (H) coinjection of dn-Xcad-11 led to no change of direction. Scale bar, 50 µm. (TIF) [file pone.0085717.s003.tif]

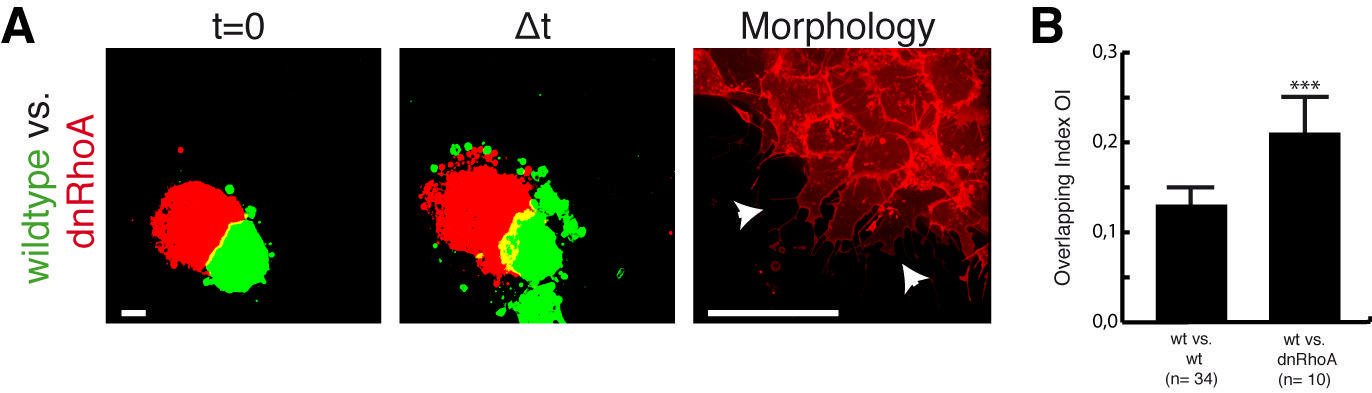

Supplement: Figure S4 — Loss of RhoA activity leads to loss of CIL. (A, B) Confrontation assay. First column: Confronted explants at time point t=0. Second column: Confronted CNC explants at time point of highest invasion Δt. Third column: Morphology of CNC cells. (A) dnRhoA led to an increased yellow overlapping area, whereas protrusion formation was same as wildtype (white arrowheads). (B) Average Overlapping Index (OI) with n = number of confrontations (wt: wildtype). Error bar shows standard error. (***) Significance to wildtype vs. wildtype with p<0.005 after student’s T-Test. Scale bar, 50 µm. (TIF) [file pone.0085717.s004.tif]
